# OmixLitMiner: A Bioinformatics Tool for Prioritizing Biological Leads from ‘Omics Data Using Literature Retrieval and Data Mining

**DOI:** 10.3390/ijms21041374

**Published:** 2020-02-19

**Authors:** Pascal Steffen, Jemma Wu, Shubhang Hariharan, Hannah Voss, Vijay Raghunath, Mark P. Molloy, Hartmut Schlüter

**Affiliations:** 1 Bowel Cancer and Biomarker Research, Kolling Institute, The University of Sydney, Sydney 2065, Australia; pascal.steffen@sydney.edu.au (P.S.); sharihar@myune.edu.au (S.H.); 2 Department of Molecular Sciences, Macquarie University, Sydney 2109, Australia; jemma.wu@mq.edu.au; 3 Mass Spectrometric Proteomics Group, Department of Clinical Chemistry and Laboratory Medicine, University Medical Center Hamburg-Eppendorf (UKE), Hamburg 20246, Germany; ha.voss@uke.de; 4 Sydney Informatics Hub, The University of Sydney, Sydney 2008, Australia; vijay.raghunath@sydney.edu.au

**Keywords:** proteomics, genomics, data mining, literature retrieval, bioinformatics, mass spectrometry

## Abstract

Proteomics and genomics discovery experiments generate increasingly large result tables, necessitating more researcher time to convert the biological data into new knowledge. Literature review is an important step in this process and can be tedious for large scale experiments. An informed and strategic decision about which biomolecule targets should be pursued for follow-up experiments thus remains a considerable challenge. To streamline and formalise this process of literature retrieval and analysis of discovery based ‘omics data and as a decision-facilitating support tool for follow-up experiments we present OmixLitMiner, a package written in the computational language R. The tool automates the retrieval of literature from PubMed based on UniProt protein identifiers, gene names and their synonyms, combined with user defined contextual keyword search (i.e., gene ontology based). The search strategy is programmed to allow either strict or more lenient literature retrieval and the outputs are assigned to three categories describing how well characterized a regulated gene or protein is. The category helps to meet a decision, regarding which gene/protein follow-up experiments may be performed for gaining new knowledge and to exclude following already known biomarkers. We demonstrate the tool’s usefulness in this retrospective study assessing three cancer proteomics and one cancer genomics publication. Using the tool, we were able to corroborate most of the decisions in these papers as well as detect additional biomolecule leads that may be valuable for future research.

## 1. Introduction

Omics analyses, regardless of the underlying acquisition platform, are united by the common feature of describing large numbers of biomolecules relevant to the experimental system under investigation. Various reporting strategies applying statistical frameworks are used in an attempt to draw out those biomolecules which are expected to be the most relevant, and hence the focus of more intense follow-up investigations. This is an important step for the researcher as assimilated understanding of experimental data through subsequent literature review is arguably the most important part of a research project, and therefore is also one of the most time-consuming tasks. Moreover, identifying relevant biomolecules from discovery studies for follow-up validation is especially important given the time to be devoted to such a task (for example, generation of a genetically engineered animal or clinical intervention). This process of literature research involves developing extensive background knowledge on the subject by searching literature databases (e.g., PubMed (https://www.ncbi.nlm.nih.gov/pubmed), Google Scholar (https://scholar.google.com) or Scopus (http://www.scopus.com).

The use of automated literature search tools is often referred to as text or data mining [1], and has been recently reviewed in a biomedical context [2]. Such tools provide a structured manner to formalise data analysis and can minimise human input errors, which might occur during manual searches. There are several different tools which have been reported for literature mining. Most of these tools use text mining for improved annotation of a target protein or gene list [3], to extract protein–protein interactions [4] or predict gene-gene relationships [5]. Other tools such as MedMiner [6], which represents one of the first instances where data mining was used in an ‘omics approach, is outdated and unsupported. More recently, Yu et al. [7] and Lau et al. [8] reported literature retrieval tools based on distinct keyword searches (i.e., gene ontology terms) to support development of targeted workflows such as multiple-reaction-monitoring (MRM). These tools generate target lists without a priori consideration of the project background/objective and without reference to acquired ‘omics discovery data. These tools are distinctly different from our report where we have developed a ‘reverse’ solution to strategy described above [7,8].

The use of automated literature search tools is often referred to as text or data mining [1], and has been recently reviewed in a biomedical context [2]. Such tools provide a structured manner to formalise data analysis and can minimise human input errors, which might occur during manual searches. There are several different tools which have been reported for literature mining. Most of these tools use text mining for improved annotation of a target protein or gene list [3], to extract protein–protein interactions [4] or predict gene-gene relationships [5]. Other tools such as MedMiner [6], which represents one of the first instances where data mining was used in an ‘omics approach, is outdated and unsupported. More recently, Yu et al. [7] and Lau et al. [8] reported literature retrieval tools based on distinct keyword searches (i.e., gene ontology terms) to support development of targeted workflows such as multiple-reaction-monitoring (MRM). These tools generate target lists without a priori consideration of the project background/objective and without reference to acquired ‘omics discovery data. These tools are distinctly different from our report where we have developed a ‘reverse’ solution to strategy described above [7,8].

We set out to devise a literature retrieval and ranking strategy of acquired ‘omics data in order to streamline data review and prioritize follow-up studies. This remains a bottleneck in ‘omics studies as often hundreds of proteins/genes are returned as significant and identifying how to proceed with such data can be daunting. The automated literature search uses a strict publication search which involves considering the biomolecule of interest (gene/protein) as well as a contextual keyword expected in the article title and/or abstract. This ensures that prior research was specifically aimed at the molecule of interest. We used this concept to build OmixLitMiner which is a bioinformatics R package that automates literature review for prioritization of biological leads from ‘omics discovery data. As input, the tool uses either the UniProt Accession number or gene names, their corresponding taxonomy identifier and keyword/s. It then automatically collects the gene name with any synonyms present in the UniProt repository to use these in the search, a clear advantage over manual searching. Furthermore, it strictly uses only reviewed UniProt entries to obtain the highest degree of confidence for search results. Using these variables, the tool constructs a query term for a PubMed search where one can specify to only look for these variable terms in the title (strictest search) or allow for one, or all query terms to be found in the abstract (more lenient search). The tool then categorises papers based on publication frequency and type as a proxy for perceived community relevance. The output can then be readily assessed to inform subsequent validation/future studies. We examined the utility of OmixLitMiner by selecting two of our previous cancer proteomics publications, which had detailed documentation of their datasets, and an independently produced cancer proteomics publication. We assessed whether the tool retrieved the same or provided additional biological lead molecules as discussed in the respective papers. We also show the tool’s utility to work with genomics data using gene names as data input.

## 2. Results

**Figure 1:**
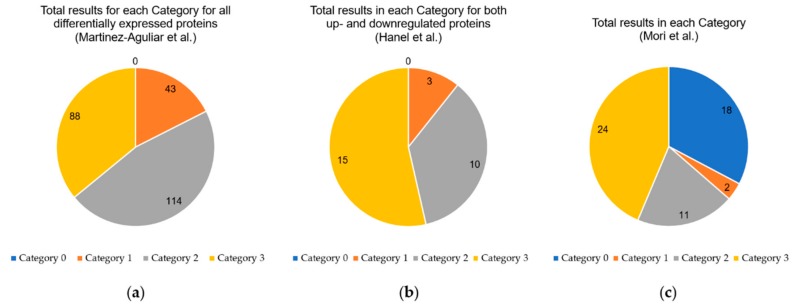
Distribution of the different categories for all proteins analysed by the OmixLitMiner tool. (**a**) The results of Martinez-Aguilar et al. [9]; (**b**) the results of Hanel et al. [10]; (**c**) the results of Mori et al. [11]. Category 1: review articles found; Category 2: original papers found, but no reviews; Category 3: no articles found; Category 0: protein absent in reviewed UniProt database (see Section 4.2 for details).

The objective of OmixLitMiner is to assist the researcher reduce the time spent on literature research of ‘omics-generated data by automating relevant literature retrieval and categorizing the results for follow-up analysis. The results from OmixLitMiner should provide the researcher with a thorough overview of the relevant literature for the biomolecules of interest (proteins in this case) in a specific context (keyword). To demonstrate and evaluate OmixLitMiner we applied it to data from three publications which used proteomic mass spectrometry analysis in a context of cancer biomarker analysis. The automated outputs from OmixLitMiner are shown as Supplementary Files S1–S12 and manually extracted summaries from these as Table 1, Table 2 and Table 3. A graphical overview of the categorisation of retrieved literature from OmixLitMiner analysis of the three publications is shown in Figure 1. For a description of the categorization terminology, see Section 4.2 in the Materials and Method section.

To evaluate OmixLitMiner we first examined the data set from Martinez-Aguilar et al. [9] who examined three subtypes of thyroid tumours using SWATH-MS. iTRAQ and MRM experiments were also conducted for validation. As this was an extensive, multi-group study we focused the assessment of OmixLitMiner on the differentially expressed proteins observed between normal thyroid tissue and the three tumour subtypes determined from the SWATH analysis (Supplementary Files S1–S3). For this publication, we first chose the keyword ‘thyroid’, but it reported only minimal results (see Supplementary File S4). Therefore, we broadened the search by using the keyword ‘Cancer’.

The proteins shown in Table 1 were some of the prominent ones selected by Martinez-Aguilar et al. [9] for further discussion. They placed particular emphasis on decorin as this showed over 10-fold reduced levels in follicular-patterned tumour subtype compared to normal thyroid tissue. OmixLitMiner analysis for decorin yielded two papers and placed it in Category 2. Interestingly, both papers were published after the publication by Martinez-Aguilar et al. [9], retrospectively justifying their interest in decorin. A similar finding was reported for superoxide dismutase 3 (*SOD3*), as two of the three reported papers were published subsequent to the Martinez-Aguilar et al. [9] paper. Another protein of particular interest to Martinez-Aguilar et al. [9] was *TGFBI*. The OmixLitMiner search yielded 18 papers, including one review article, thus placing it in Category 1. Five of these papers were published subsequent to Martinez-Aguilar et al. [9], and one papers was placed in the ‘False’ column as assigned by the script. Here, the ‘False’ flag represents a check where there is missing information in the automated PubMed returned metadata, requiring these results to be manually curated. It is likely that the relatively large change in abundance of this protein and the extensive literature on this protein prompted Martinez-Aguilar et al.’s [9] interest in this protein. An interesting finding was for the search for Actin B (*ACTB*), as it was placed in Category 1, yet only yielded four papers. This is due to actin B being used as reference (‘housekeeping’) gene in some cancers because of its often stable and high expression [12] and thus often appears in conjunction with cancer in publication titles (this is the case for 3 of the 4 hits OmixLitMiner reported, see Supplementary File S4). Another interesting result was that our search for actin-related protein 2/3 complex subunit 5 (*ARPC5*), did not return any results. When looking into *ARPC5* we found that it only represents a single subunit of the *ARP2/3* complex, and thus may be unlikely to be included in the title of publications, and hence may have evaded our search.

As shown in Figure 1, 114 of the 245 (46.5%) differentially expressed proteins had Category 2 hits, whilst 43 (17.6%) had Category 1. This indicates that many of the proteins with the most significant differential expression have already been the focus of previous reports in various cancers.

We also looked at proteins showing Category 1 and 2 hits which were not specifically discussed in the publication of Martinez-Aguilar et al. [9]. For this analysis we limited the data to the normal vs. papillary thyroid subtype SWATH dataset (184 significantly regulated proteins). One hundred and thirteen (113) proteins were Category 1 or 2 hits in our search. For these 113 proteins we looked at the publication dates OmixLitMiner found and removed those that were published after the submission date of Martinez-Aguilar et al. [9] (07.09.2015) which left a list of 21 in Category 1 and 71 in Category 2 (see Supplementary File S5 for the filtered list). Clearly, with so many articles retrieved by OmixLitMiner we could readily find interesting proteins which escaped attention of Martinez-Aguilar et al. [9]. For example, *GPX3* is a Category 2 protein where suppression directly correlates with promotion of metastasis in human thyroid cancer [13], and the Category 1 protein, *BAX* has been reported to be upregulated in relation to papillary thyroid cancer [14]. We point out these examples simply to demonstrate the utility of OmixLitMiner to find interesting lead proteins for future study.

We next examined a dataset by Hanel et al. [10], who used a mouse xenograft model to investigate proteins that facilitate metastasis. Using a mouse model with human neuroblastoma xenograft, they analysed samples from both liver and ovarian metastases using LC-MS/MS and compared it to the primary xenograft tumour. For our analysis using OmixLitMiner, we selected the proteins that were differentially expressed at both distant sites compared to the primary tumour. Table 2 contains some of these proteins, which were selected for further discussion by Hanel et al. [10]. We initially used the search keyword ‘Metastasis’, but this yielded only Category 3 hits (*LGALS7* was an exception, Category 2), so we reverted to the broader term “Cancer”. See Supplementary Files S6 and S7 for the complete results.

As shown in Table 2, acyl-protein thioesterase 2 (*LYPLA2*), peripherin (*PRPH*) and neurofilament medium polypeptide (*NEFM*) did not yield any results. This is likely because Hanel et al. [10] searched their mass spectra against a database containing human as well as mouse protein sequences. As a result, when OmixLitMiner searched for these proteins using mouse taxonomy identifier, no results were returned, due to a lack of literature concerning these mouse proteins. Since a mouse model was used, it is unclear if the inclusion of these proteins was accidental or intentional. We investigated this by a manual search of PubMed and found the same results confirming the problem was not a result of an error in the OmixLitMiner script. In our experience, such a result seems to occur very rarely, despite a seemingly correct PubMed query. As OmixLitMiner cannot overcome this PubMed error, our tool flags such an occurrence by using the “False” column.

Table 2 contains two proteins with true Category 2 hits. Eukaryotic translation initiation factor 4B (*EIF4B*) had two papers, both of which were published before the Hanel et al. [10] paper. Similarly, protein DPY-30 (*DPY30*) also had two papers, only one of which was published prior to Hanel et al. [10]. Hence, we would agree that these proteins would be interesting candidates for further investigations. Peripherin-2 (*PRPH*) did not return any results and was hence placed in Category 3. As our search did not return any publications for this protein, the protein may indeed be a novel and promising candidate for future study, but the lack of current corroborating literature suggests caution in executing follow-up studies.

Finally, we reviewed the article by Mori and colleagues [11] who investigated putative protein markers associated with lymph node metastasis in colorectal cancer. They used iTRAQ labelling and mass spectrometry for discovery of candidate proteins, then through data filtering arrived at the protein Ezrin, which they focused on for validation using IHC and qRT-PCR. Mori et al.’s [11] strategy was to filter the iTRAQ results based on statistical analysis of differential ratios of abundance, identifying 55 proteins. They then used functional annotations to select four proteins which were part of metastatic processes and finally selected Ezrin for validation because of on its high corresponding differential mRNA level determined by qRT-PCR.

For our analysis using OmixLitMiner we selected the list of 55 proteins that passed Mori et al.’s [11] initial filtering threshold and used the search keyword “metastasis”. The output from OmixLitMiner are reported in Supplementary File S8. To simplify our evaluation, we extracted the results from OmixLitMiner for the four proteins (ezrin, interleukin enhancer-binding factor 3, tropomyosin alpha-3 chain and keratin, Type I cytoskeletal 18) which were the focus of Mori et al.’s [11] discussion and presented them here in Table 3.

As can be seen in Table 3 half the proteins mentioned by Mori et al. [11] were assigned by OmixLitMiner into Category 2, which means that at least one publication but no review article was found in combination with the selected keyword in a publication title. We consider that Category 2 hits are generally preferred for further analysis as they can be verified by existing literature but have typically not been extensively investigated in the selected context to warrant biomolecule-specific review articles. Such proteins provide good opportunity to discover new knowledge from subsequent follow-up experiments. For the two Category 2 hits only one publication for *EZR* and two publications for *ILF3* were found in context with metastasis. Interestingly, the article retrieved for the ezrin search was published subsequently to Mori et al. [11], corroborating that finding. Of the remaining three proteins, *ILF3* was a Category 2 hit with two publications, while *KRT18* was a Category 3 hit, meaning no publication in context with the keyword was found. The reported UniProt accession number for *TMP3* corresponded to its isoform 2 and was therefore excluded by OmixLitMiner. This can be easily seen as it was assigned to Category 0. Mori et al. [11] similarly only made fleeting reference to these proteins. Overall, we feel that the results from OmixLitMiner are in agreement with the proteins selected for discussion by Mori et al. [11] regarding the most promising targets for further analysis as they were Category 2 hits.

OmixLitMiner is also compatible with genomics data. We demonstrated this with an analysis of a publication from Tian et al. [15], who detected a 13 panel gene signature for colorectal cancer recurrence. The input as well as output files from OmixLitMiner are shown in Supplementary Files S9–S12.

## 3. Discussion

We report the development of a bioinformatics tool, OmixLitMiner, which harvests relevant literature from PubMed to assist ‘omics researchers in prioritising leads for follow-up from large-scale ‘omics discovery experiments. The tool is provided as a ready to install R package and categorises previous literature into three groups based on publication frequency, or in other words, the perceived community-determined importance of specific biomolecules in a particular biological context. We use “review articles” as a proxy for likely importance of a biomolecule target. This can be helpful in excluding target biomolecules which have yet to receive sufficient contextual importance to warrant a review. Follow-up of targets from this category might be considered higher-risk. Conversely, Category 1 hits are likely to be judged to be important, but could be deemed to be too widely studied, making it difficult to generate new insightful impact from follow-up and validation studies. Of course, such categorisation is also a potential weakness, as interesting, unstudied biomolecules may be excluded on such basis if one solely relies on this categorization and completely discards Category 3 hits. Clearly, it remains the domain of the researcher to decide on a case to case basis which proteins are deemed to be worthy of further study.

We demonstrated the utility of OmixLitMiner by evaluating its output from three published articles from the cancer proteomics literature. Overall, we showed that OmixLitMiner was generally concordant with reporting proteins which were a focus of discussion within the corresponding papers. Additionally, we were able to determine that interesting unreported aspects of studies, such as Mori et al. [11] using a TrEMBL FASTA database which included isoforms and Hanel et al. [10] reporting mouse proteins in their final results from analysis of human cancers grown in mouse models. While looking through parts of Martinez-Aguilar et al. [9] data set we found many additional proteins which might be of interest for follow-up in the context with thyroid cancer. These examples demonstrate the power of such a tool even when used retrospectively.

We demonstrated the utility of OmixLitMiner by performing comparisons with several published articles. While we did not conduct formal user-experience testing, the analysis reported here was conducted by a bachelor level student with no prior participation with any of the papers, suggesting the tool could be readily used by following our SOP located on the GitHub repository. This suggests that even with minimal prior background knowledge (which might often be the case when running discovery-based experiments for a collaborator) the results of the OmixLitMiner tool can rapidly prioritise ‘omics discovery leads.

The authors are also aware that the parameters used in this paper (synonym and keyword must be in the title) are very strict and therefore potentially exclude important literature which may be found using more sophisticated informatics approaches [2]. It is important that the user explore keyword terms, which would be required in a manual search anyway. The tool also does not include any sophisticated machine learning or false-discovery algorithms, but rather retrieves a raw output of the search results in the space of the selected input and keywords (superordinate search) and scores each gene/protein accordingly. The main reason for this is that the authors believe that it is of utmost importance that as a last step the researchers needs to assess the presented data by themselves for the most optimal results. We feel that the time saved by giving the researcher access to a complete summary table with a simple categorizing system is beneficial to assist researchers extract useful biological knowledge from their ‘omics experiments.

## 4. Materials and Methods

### 4.1. Literature Retrieval and Ranking

OmixLitMiner is composed of three major components: query building, query retrieval text analysis and ranking. It utilises two major publicly available biomedical repositories: UniProt [16] and NCBI PubMed databases [17]. OmixLitMiner is freely available on the GitHub repository (https://github.com/Sydney-Informatics-Hub/OmixLitMiner).

The input to OmixLitMiner can be a single query or list of queries in an Excel file or dataframe. Each row represents a query and each query consists of a protein identifier (UniProt accession) or gene name, the NCBI species taxonomy identifier (e.g., 9606 for *Homo sapiens*), a list of keywords of interest (e.g., prostate cancer) and an indicator specifying if the search keywords appear in article titles only, or abstract or title. Each query will be processed independently by OmixLitMiner. To improve the coverage of the query, OmixLitMiner utilises all available gene synonyms recorded in UniProt and builds expanded queries. OmixLitMiner exploits the UniProt REST web service to fetch the synonyms for a given UniProt and taxonomy identifier. After fetching the synonyms, an expanded PubMed query is built, which includes the protein/gene names and synonyms, taxonomy identifier, keywords as well as its search filter. The query can also optionally include the publication date range to further focus the interested literature.

After building the expanded PubMed query, OmixLitMiner executes automated document retrieval from the PubMed database using these queries. OmixLitMiner uses function EUtilsSummary and EUtilsGet in the RISmed R package [18] to automatically download the abstracts and metadata from the PubMed database. The metadata includes title, authors, country, publication year, MeSH (Medical Subject Headings) terms and article type (review or not).

The automated retrieved PubMed records undergo statistics and text analysis to yield summarisation, clustering and categories of the retrieved results. Visual plots including barplots of the publications and MeSH terms are produced which can reveal the distribution and trends of the research. The word cloud of abstracts provides a visualisation of the text data and gives a highlight of the most frequently appeared words in all the abstracts. MeSH terms are a controlled vocabulary for medical research and most of PubMed records are tagged with a set of these terms. OmixLitMiner generates a MeSH-based clustering for all the retrieved records. The clustering adopts a hierarchical clustering method with cosine distance, which is defined as $\cos\left(A,B\right)=\;\frac{AB}{\left|\left|A\right|\right|\left|\left|B\right|\right|}$ where A and B are two MeSH matrices; the default number of clusters is 4. This clustering method provides a way of organising all the retrieved records by putting together the documents with similar research topics. Besides unsupervised clustering, the results are classified into three pre-defined categories.

**Figure 2:**
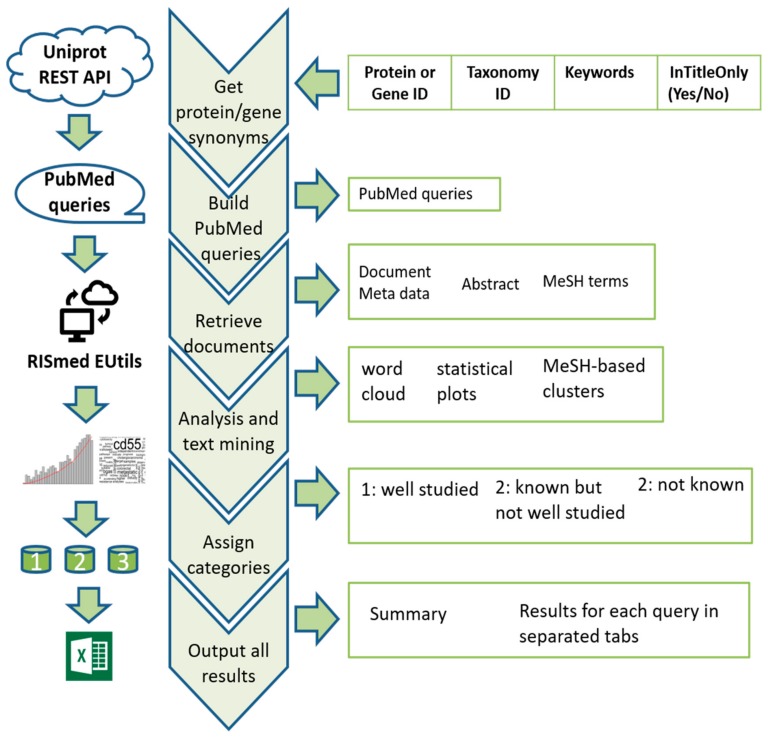
Schematic workflow showing the different steps the OmixLitMiner tool goes through before generating an output file.

The retrieved PubMed abstracts and metadata are output into a separate tab for each query in an Excel file, along with the accompanied analysis results. A summary table for all query results is generated and output in the first tab of the file. Figure 2 shows the schematic workflow of the OmixLitMiner tool.

### 4.2. Categorization of the Results

The tool assigns the proteins into three main categories (1–3) and an additional Category 0. Category 1 hits are proteins/genes which show at least one review paper where the synonyms and the selected keywords are found together in the article title, or in the abstract if that option is selected. Category 2 hits are proteins/genes where at least one publication was found, but no review article, in which the synonyms and the selected keywords are both present. Category 3 represents proteins/genes where no publication was found which mentions both the synonyms and the keywords together in the title. Category 0 is used for proteins/genes where the tool could not find any synonyms. This may happen if the UniProt accession belongs to an isoform or to an entry that is unreviewed (i.e., TrEMBL), see Supplementary File S8. The tool also conducts a check to see if the keyword along with one of the synonyms is in the title (or abstract). If this check is negative, then the hit is categorized as “False”. In our experience this happens only rarely and is caused by missing information in the automated PubMed returned metadata.

### 4.3. Evaluation Strategy

To demonstrate the tool’s performance, we selected ‘omics publications with detailed Supplemental data relevant to cancer biomarker discovery. This included two proteomics publications (Martinez-Aguliar et al. [9] and Hanel et al. [10]), and an independent proteomics publication (Mori et al. [11]). For each publication the most significant hits as discussed in the results of each paper where chosen for analysis. The UniProt accession numbers, specific keyword (cancer or metastasis) and the corresponding taxonomy ID where copied into an Excel input file for analysis (Supplementary Files S1–S12). The tool was run using R 3.5.1. The OmixLitMiner analysis was performed by a bachelor student (S.H.) who had no involvement in the original publications. The presented results were obtained on 13.03.2019 and are expected to change as the PubMed library grows. We also included OmixLitMiner search results from a cancer genomics publication [15] to demonstrate the tool’s compatibility with this data type.

## Figures and Tables

**Table 1:** Extract of the OmixLitMiner output of the three comparisons found in the supplement of Martinez-Aguilar et al. [9] using the keyword ‘cancer’.

Keyword: Cancer						
Protein		Summary from Martinez-Aguilar et al. [9]	OmixLitMiner results			
UniProt ID	Gene Name		Total	Category	False	Comments
Q15582	*TGFBI*	Was elevated in 6/8 FTC samples compared to FA samples. Interested in it as a potential marker for the progression of adenoma to malignancy in follicular thyroid tumours	18	1	1	
P12830	*CDH1*	PTC tumours express lower levels of E-cadherin	204	1	4	
Q07654	*TFF3*	Dysregulated in both PTC and FTC (compared to normal)	22	1	1	6 papers were published after Martinez-Aguilar
P60709	*ACTB*	Overexpressed in PTC, associated with actin cytoskeleton remodeling	4	1	0	One paper was published after Martinez-Aguilar
P07585	*DCN*	Decorin was lost in both FA and FTC, but not PTC. Decorin expression supposedly decreases metastasis	2	2	0	Both papers were published after Martinez-Aguilar
P08294	*SOD3*	Dysregulated in both PTC and FTC (compared to normal)	3	2	0	One paper was published after Martinez-Aguilar
O15511	*ARPC5*	Overexpressed in PTC, associated with actin cytoskeleton remodeling	0	3	0	

**Table 2:** Extract of the OmixLitMiner output of the differentially expressed proteins at both distant sites compared to the primary tumour as reported in the supplement of Hanel et al. [10] using the keyword ‘Cancer’.

Keyword: Cancer						
Protein		Summary from Hanel et al. [10]	Our Results			
UniProt ID	Gene Name		Total	Category	False	Comments
P23588	*EIF4B*	Was strongly upregulated at both sites, and is upregulated by a downstream signaling cascade initiated by *LYPLA2*	2	2	0	
Q9C005	*DPY30*	Was strongly upregulated at both sites	2	2	0	One publication after Hanel et al. [10]
P47929	*LGALS7*	Was strongly downregulated at both sites	0	3	0	
Q9WTL7	*LYPLA2*	Was upregulated at both sites. The paper has also recognised that it is currently under investigation as a potential anti-cancer target	0	3	0	This version of the LYPLA2 protein is found in mice, not humans. As a mouse model was used in their investigation, its inclusion may or may not have been intentional
P41219	*PRPH*	Was downregulated at both sites	0	3	0	
P08553	*NEFM*	Was strongly downregulated at both sites, though its involvement in metastasis is unclear	0	3	0	This version of the NEFM protein is found in mice, not humans.

**Table 3:** Extract from OmixLitMiner from the list of 55 proteins reported in the supplement of Mori et al. [11] using the keyword “Metastasis”.

Keyword: Metastasis						
Protein		Summary from Mori et al. [11]	OmixLitMiner Results			
UniProt ID	Gene Name		Total	Category	False	Comments
P15311	*EZR*	Was prominently associated with metastasis and is functionally involved in the ‘development process’. mRNA expression was also elevated in CRC with LN Metastasis	1	2	0	Published subsequent to Mori et al. [11]
Q12906	*ILF3*	Was prominently associated with metastasis and is functionally involved in the ‘development process’	2	2	0	
P06753-2	*TPM3*	Was prominently associated with metastasis and is functionally involved in the ‘development process’	11	0	11	This is one of the isoforms of *TPM3* and is thus not considered by the script.
P05783	*KRT18*	Was prominently associated with metastasis and is functionally involved in the ‘development process’	0	3	0	

## Supplementary Materials

Supplementary materials can be found at https://www.mdpi.com/1422-0067/21/4/1374/s1.

## Abbreviations

MRM	Multiple-Reaction-Monitoring
SWATH-MS	sequential window acquisition of all theoretical fragment ion spectra mass spectrometry
iTRAQ	Isobaric tags for relative and absolute quantitation
qRT-PCR	Real-Time Quantitative Reverse Transcription PCR
